# Identifying observational studies of surgical interventions in MEDLINE and EMBASE

**DOI:** 10.1186/1471-2288-6-41

**Published:** 2006-08-18

**Authors:** Cynthia Fraser, Alison Murray, Jennifer Burr

**Affiliations:** 1 Health Services Research Unit, University of Aberdeen, Health Sciences Building, Foresterhill, Aberdeen AB25 2ZD, UK

## Abstract

**Background:**

Health technology assessments of surgical interventions frequently require the inclusion of non-randomised evidence. Literature search strategies employed to identify this evidence often exclude a methodological component because of uncertainty surrounding the use of appropriate search terms. This can result in the retrieval of a large number of irrelevant records. Methodological filters would help to minimise this, making literature searching more efficient.

**Methods:**

An objective approach was employed to develop MEDLINE and EMBASE filters, using a reference standard derived from screening the results of an electronic literature search that contained only subject-related terms. Candidate terms for MEDLINE (N = 37) and EMBASE (N = 35) were derived from examination of the records of the reference standard. The filters were validated on two sets of studies that had been included in previous health technology assessments.

**Results:**

The final filters were highly sensitive (MEDLINE 99.5%, EMBASE 100%, MEDLINE/EMBASE combined 100%) with precision ranging between 16.7% – 21.1%, specificity 35.3% – 43.5%, and a reduction in retrievals of over 30%. Against the validation standards, the individual filters retrieved 85.2% – 100% of records. In combination, however, the MEDLINE and EMBASE filters retrieved 100% against both validation standards with a reduction in retrieved records of 28.4% and 30.1%

**Conclusion:**

The MEDLINE and EMBASE filters were highly sensitive and substantially reduced the number of records retrieved, indicating that they are useful tools for efficient literature searching.

## Background

When assessing the safety and efficacy or effectiveness of health technologies it may not be appropriate to restrict the evidence to randomised controlled trials (RCTs) [1]. There are many situations where it may be considered necessary to include evidence from observational studies such as non-randomised comparative and case series studies [1]. In a survey of health technology assessments undertaken for the UK National Institute for Health and Clinical Excellence (NICE), 30% were found to include case series evidence [1]. A review of the included evidence for six systematic reviews, commissioned by NICE for their Interventional Procedures Program (IPP), found that 86% of the data came from case series with less than 5% from RCTs [2]. The most common reason given for inclusion of case series data was the absence of sufficient data from randomised evidence to assess effectiveness or safety outcomes. Indeed, in some cases, it was the only form of evidence available.

Searching for randomised evidence is relatively straightforward with the introduction of several initiatives to aid retrieval: the CENTRAL database of trials in The Cochrane Library; appropriate indexing terms in MEDLINE and EMBASE; and published highly sensitive filters [3-6]. There is also some evidence that the introduction of the CONSORT statement is associated with better reporting of randomised trials in the titles and abstracts and hence facilitates both searching and indexing [7].

Searching for non-randomised evidence of safety and efficacy or effectiveness from primary studies, however, is more problematic and there has been little published research to date. Indexing terms are less well established [8] and, when they do exist, are used inconsistently [9]. The reporting of methodological detail is often poor in observational studies and this contributes to problems in indexing and searching effectively [1].

The uncertainty in identifying appropriate search terms for non-randomised evidence has meant that a methodology component is often excluded from search strategies. This can lead to an inefficient use of valuable resources in terms of time involved in screening the titles and abstracts of a large number of irrelevant records. For the purposes of this study, health technology assessments and systematic reviews commissioned by NICE and published on their website at October 2005 were surveyed. 77 health technology assessments from the main NICE program and the seven systematic reviews carried out for the IPP programme were reviewed. 28 (36.4%) of the technology assessments and seven (100%) of the IPP reviews included non-randomised evidence. 31(88.6%) of these reports used no methodology filter in their search strategies while two reviews of diagnostic interventions used diagnostic filters and two strategies used adverse events filters to identify supplementary safety data.

Efficient searching for the evidence, for health technology assessments, requires an effective filter. The filter should maintain the sensitivity (or recall) of the original subject-only search, retrieving the same relevant studies (cell A of Table 1). In addition, the filter should reduce the number of irrelevant records retrieved (decrease in cell B and increase in cell D of Table 1), and hence increase precision and specificity of the search. While precision measures the number of relevant retrievals (A) in terms of the total number retrieved (A+B), the specificity measures the proportion of the non-reference standard (B+D) that are not retrieved (D). An effective filter, then, would maintain the sensitivity of the original subject search while maximising precision and specificity.

**Table 1:** Definitions of Sensitivity, Precision and Specificity

		Manual review of subject only electronic search	
		
		Non-randomised studies (Reference standard)	Other studies (Non-reference standard)
Search filter	Retrieved	A	B
	Not retrieved	C	D

Sensitivity = A/(A+C) × 100

Precision = A/(A+B) × 100

Specificity = D/(B+D) × 100

The aim of this study was to develop effective MEDLINE and EMBASE filters, to identify non-randomised evidence for surgical interventions, to be used in conjunction (using Boolean operator AND) with a subject search strategy.

## Methods

### Establishment of a reference standard

In a recent systematic review to assess the effectiveness and safety of laser in-situ keratomileusis (LASIK), the MEDLINE and EMBASE search strategies used to identify the evidence had incorporated terms that pertained only to the intervention and medical conditions of interest to the review and were restricted to the publication years 2000–2004 [10]. The MEDLINE and EMBASE strategies were run simultaneously as a multi-file search in Ovid and the results de-duplicated using the Ovid de-duplication tool. The retrieved titles and abstracts were screened and all reports within the scope of the topic that appeared to be randomised controlled trials, non-randomised comparative studies and case series were identified. Both prospective and retrospective studies were included. The full papers of these reports were acquired and study design assessed by one experienced reviewer. Any uncertainty was resolved by consultation with another reviewer involved in the review. The non-randomised studies constituted the reference standard for this study, against which the new methodology filters could be developed and assessed. (Subsequently for the purposes of the systematic review, smaller non-randomised and case series studies were excluded.)

### Identification of the candidate terms for the filters

The titles, abstracts, thesaurus controlled subject headings (for MEDLINE and EMBASE) and the publication type field (for MEDLINE) were subjectively assessed by the information specialist for all the MEDLINE and EMBASE records of the reference standard. Terms that explained or gave an indication of methodology employed or systematic assessment of postoperative sequelae were identified and considered as candidate terms for the MEDLINE and EMBASE methodology filters. By incorporating each term individually with the original MEDLINE or EMBASE subject search strategy (using the Boolean operator AND), the sensitivity (proportion of reference standard retrieved); precision (proportion of total retrieved that were included in the reference standard); and specificity (proportion of non-reference standard studies that were not retrieved) of the candidate terms were calculated.

### Development of the filters

Initially all the candidate terms for each database were combined, using the Boolean operator OR, to form the separate MEDLINE and EMBASE filters. These were run in combination with the subject-only search strategies (using the Boolean operator AND). Each candidate term was then tested to establish if its removal from the filter reduced overall sensitivity. If sensitivity was unaffected the term was considered redundant and was excluded from further analysis, while if sensitivity decreased the term was re-instated. To minimise the number of irrelevant records retrieved, two approaches were explored:

1. The terms were tested in order of precision, beginning with the lowest so that preference was given to retaining the terms with higher precision. The resulting MEDLINE and EMBASE filters are referred to as the *Precision Terms Filters*.

2. The terms were tested in order of specificity, beginning with the lowest so that preference was given to retaining the terms with higher specificity. The resulting MEDLINE and EMBASE filters are referred to as the *Specificity Terms Filters*.

By this process of elimination, redundant terms were removed and the combination of retained terms aimed to minimise the number of retrieved irrelevant records. Four filters were thus developed: *Precision Terms Filters *for both MEDLINE and EMBASE, and *Specificity Terms Filters *for both MEDLINE and EMBASE.

### Assessing the performance of the filters

The subject-only MEDLINE and EMBASE search strategies were run with (using Boolean operator AND) and without the resulting filters. The total number of records and the number of reference standard records that were retrieved were used to calculate the retrieval parameters. The *Specificity Terms Filters *for MEDLINE and EMBASE and the *Precision Terms Filters *for MEDLINE and EMBASE were also run simultaneously in multi-file MEDLINE and EMBASE searches, the results de-duplicated using the Ovid de-duplication tool, and retrieval parameters calculated.

### Validation of the filters

The performances of the preferred filters were tested against two validation standards. These comprised the included non-randomised studies from two other reviews: a systematic review of photorefractive keratomileusis (PRK) for myopia [10] and electrosurgery for tonsillectomy [11]. The original MEDLINE and EMBASE strategies used to find the evidence for these reviews were subject-only searches and did not include any methodology filters. The PRK search strategy searched for publications in the years 2000–2004 and for tonsillectomy, 1990 to 2004.

The validations standards were incomplete in comparison to our reference standard because they did not include all non-randomised studies that met the inclusion criteria in terms of intervention and medical condition. Some non-randomised studies had been excluded at the screening stage because they did not fulfil other criteria, such as sample size or, in the case of the tonsillectomy review, were retrospective. These studies were not readily identifiable for inclusion in this study. The validation standards, therefore, underestimated the total number of non-randomised studies that were identified from the subject-only searches (cells A and C in Table 1) and falsely assigned these non-randomised studies to the non-validation standards (cells B and D of Table 1). Because of these false assignments it was not possible to accurately calculate the search parameters. The original search strategies were run with and without the inclusion of the new filters, and performance compared in terms of the proportion of the validation standards that were retrieved and the reduction in number of retrievals.

## Results

### Description of reference standard

The reference standard comprised 217 articles. Table 2 details the composition of the reference standard in terms of study design and database inclusion. Most studies were case series (83.9%) and the majority were assessed as prospective (56.6%). Not all were indexed in both MEDLINE and EMBASE: 206 (94.9%) in MEDLINE, 191 (92.7%) in EMBASE and 180 (82.9%) in both.

**Table 2:** Composition of Reference Standard

Study design	Total		MEDLINE		EMBASE	
	N	%	N	%	N	%
Comparative – prospective	19	(8.7)	18	(8.7)	16	(8.4)
Comparative – retrospective	16	(7.4)	14	(6.8)	13	(6.8)
Case series – prospective	104	(47.9)	99	(48.1)	93	(48.7)
Case series – retrospective	70	(32.3)	67	(32.5)	61	(31.9)
Case series – unclear	8	(3.7)	8	(3.9)	8	(4.2)

TOTAL	217	(100.0)	206	(100.0)	191	(100.0)

### Candidate terms

Table 3 lists the 37 candidate terms, identified from the 206 MEDLINE records, and their retrieval parameters. 11 controlled thesaurus terms (8 MeSH terms and 3 terms from the publication type field), 18 text words or phrases from the titles and abstracts and 8 words or phrases that occurred either in the MeSH terms, title or abstract fields were included. There was considerable variation in the performance of these terms. Sensitivity varied from 1.0% to 65.5%: the most sensitive terms, retrieving the highest proportion of the reference standard, were *(compare$ or compara$).mp *(65.5%); *(postoperat$ or post operat$).mp *and *(preoperat$ or pre operat$).mp *(63.1% and 54.4% respectively); *(preoperat$ or pre operat$).tw *and *(postoperat$ or post operat$).tw *(53.4.0% and 51.0% respectively); and the MeSH term *Comparative studies *(51.5%). Precision varied from 1.3% to 41.7% with highest values for *cohort.tw *(41.7%), *cohort.mp *(37.9%) and *(non random$ or nonrandom$).tw *(37.2%). The ability of the search terms to exclude non-reference standard articles, their specificity, was generally high, with poorest performance found for the terms *(postoperat$ or post operat$).mp *(54.2%), *(postoperat$ or post operat$).tw *(68.1%) and *evaluat$.tw *(70.6%).

**Table 3:** Candidate search terms for MEDLINE

Search Term	Reference Standard retrieved (N = 206)	Total retrieved (N = 1564)	Sensitivity	Precision	Specificity
**Controlled Thesaurus Terms**					
Case-control studies/	2	8	1.0	25.0	99.5
Cohort studies/	5	16	2.4	31.2	99.2
Comparative studies/	106	326	51.5	32.5	83.8
Follow-up studies/	35	135	17.0	25.9	92.6
Prospective studies/	68	240	33.0	28.3	87.3
Retrospective studies/	78	282	37.9	27.7	85.0
Time factors/	15	60	7.3	25.0	96.7
Treatment outcome/	75	323	36.4	23.2	81.7
Case reports.pt	5	393	2.4	1.3	71.4
Clinical trial.pt	25	139	12.1	18.0	91.6
Evaluation studies.pt	12	44	5.8	27.3	97.6
**Textwords (.tw)**					
baseline	16	49	7.8	32.6	97.6
case control$	3	10	1.5	30.0	99.5
case series	28	132	13.6	21.2	92.3
cases	40	223	19.4	17.9	86.5
chang$	56	310	27.2	18.1	81.3
cohort	10	24	4.8	41.7	99.0
compare$ or compara$	98	312	47.6	31.4	84.2
consecutive$	56	163	27.2	34.4	92.1
evaluat$	100	499	48.5	20.0	70.6
follow$	59	272	28.6	21.7	84.3
non compara$ or noncompara$	14	83	6.8	16.9	94.9
non random$ or nonrandom$	19	51	9.2	37.2	97.6
observational	3	22	1.5	13.6	98.6
post operat$ or postoperat$	105	538	51.0	19.5	68.1
pre operat$ or preoperat$	110	454	53.4	24.2	74.7
prospective$	60	216	29.1	27.8	88.5
retrospective$	66	242	32.0	27.3	87.0
reviewed	19	67	9.2	28.4	96.5
**MeSH and/or Textword (.mp)**					
case control	4	12	1.9	33.3	99.4
cohort	11	29	5.3	37.9	98.7
compare$ or compara$	135	464	65.5	29.1	75.8
follow$	68	312	33.0	21.8	82.0
prospective$	69	260	33.5	26.5	85.9
retrospective$	79	296	38.3	26.7	84.0
postoperat$ or post operat$	130	752	63.1	17.3	54.2
preoperat$ or pre operat$	112	466	54.4	24.0	73.9

Key

/ MeSH term

pt Term from publication type field

tw Text word(s) from title and/or abstract fields

mp Term from MeSH and/or title and/or abstract fields.

$ Truncation symbol

The performances of the 35 candidate terms for the EMBASE records are listed in Table 4. Ten EMTREE terms, 18 textwords or phrases from the titles and abstracts and seven words or phrases that occurred in either the EMTREE terms, title or abstract fields were included. Sensitivity ranged from 0.5% to 64.4% with *(postoperat$ or post operat$).mp *(64.4%), *(preoperat$ or pre operat$).mp *(55.5%) and the subject term *Major clinical study *(55.5%) retrieving the highest proportion of the reference standard. Precision varied from 0.3% to 42.1%: the most precise being, as with MEDLINE, *cohort.tw *(42.1%), *(non random$ or nonrandom$).tw *(41.3%) and *cohort.mp *(38.1%). Lowest specificity was again found for *(postoperat$ or post operat$).mp *(47.1%), *(postoperat$ or post operat$).tw *(69.7%), *(preoperat$ or pre operat$).mp *(71.1%) as well as the subject heading *Treatment outcome *(73.0%).

**Table 4:** Candidate search terms for EMBASE

Search Term	Reference Standard retrieved (N = 191)	Total retrieved (N = 1521)	Sensitivity	Precision	Specificity
**Controlled Thesaurus Terms**					
Case report/	1	348	0.5	0.3	73.9
Clinical trial/	23	131	12.0	17.6	91.9
Cohort analysis/	3	9	1.6	33.3	99.5
Comparative study/	13	39	6.8	33.3	98.0
Controlled study/	79	309	41.4	25.6	82.7
Follow-up study/	45	212	23.6	21.2	87.4
Major clinical study/	106	308	55.5	34.4	84.8
Prospective study/	22	74	11.5	29.7	96.1
Retrospective study/	29	108	15.2	26.8	94.1
Treatment outcome/	88	447	46.1	19.7	73.0
**Textwords (.tw)**					
baseline	16	47	8.4	34.0	97.7
case control$	3	9	1.6	33.3	99.5
case series	27	122	14.1	22.1	92.9
cases	38	201	19.9	18.9	87.7
chang$	51	297	26.7	17.2	81.5
cohort	8	19	4.2	42.1	99.2
compar$ or compara$	90	287	47.1	31.4	85.2
consecutive$	51	142	26.7	35.9	93.2
evaluat$	96	425	50.3	22.6	75.3
follow$	50	259	26.2	19.3	84.3
non compara$ or noncompara$	13	78	6.8	16.7	95.1
non random$ or nonrandom$	19	46	9.9	41.3	98.0
observational	3	20	1.6	15.0	98.7
postoperat$ or post operat$	91	494	47.6	18.4	69.7
preoperat$ or pre operat$	101	418	52.9	24.2	76.2
prospective$	56	195	29.3	28.7	89.5
retrospective$	61	223	31.9	27.3	87.8
reviewed	20	66	10.5	30.3	96.5
**EMTREE and/or Textword (.mp)**					
cohort$	8	21	4.2	38.1	99.0
compare$ or compara$	90	297	47.1	30.3	84.4
follow$	61	325	31.9	18.8	80.1
prospective$	57	200	29.8	28.5	89.2
retrospective$	64	233	33.5	27.5	87.3
postoperat$ or post operat$	123	827	64.4	14.9	47.1
preoperat$ or pre operat$	106	491	55.5	21.6	71.1

Key

/ EMTREE term

tw Text word(s) from title and/or abstract fields

mp Term from EMTREE and/or title and/or abstract fields.

$ Truncation symbol

The more explicit text words and phrases, used to describe study design, such as *case series*, *case control$*, *cohort*, *observational*, *non random$ *or *nonrandom$ *and *non compara$ *or *noncompara$*, were used infrequently with sensitivity values ranging from 1.5% to 14.1%. *Compare$ *or *compara$*, however, occurred in almost half of the titles and/or abstracts (47.6% MEDLINE; 47.1% EMBASE); although this term would not necessarily be used in the context of describing study design. Whether a study was prospective or retrospective was not routinely stated in the titles or abstracts, occurring in only 61.1% of MEDLINE and 63.3% of EMBASE records.

Table 5 lists the candidate terms that remained after the stepwise elimination of redundant terms. All terms from the publication type field in MEDLINE and most of the thesaurus controlled terms were excluded (MEDLINE: 6/8 for *Precision Terms *search and 5/8 for *Specificity Terms *search and for EMBASE: 7/10 and 6/10 respectively). The MEDLINE and EMBASE *Specificity Terms Filters *included the same controlled terms as in the *Precision Terms Filters *but with the addition of one term each: *Time factors *for MEDLINE and *Clinical trial *for EMBASE. There was considerable similarity in retained text words between the strategies with three common to all four search filters (*chang$, evaluat$ and reviewed*) and two text words included in three of the strategies (*baseline, compare$ or compara$)*. The term *preoperat$ or pre operat$.mp *was also included in three strategies.

**Table 5:** Retained candidate terms

**MEDLINE**		**EMBASE**	
**Precision**	**Specificity**	**Precision**	**Specificity**

Comparative studies/ Follow-up studies/	Comparative studies/ Follow-up studies/ Time factors/	Controlled study/ Treatment outcome/ Major clinical study/	Controlled study/ Treatment outcome/ Major clinical study/ Clinical trial/
(preoperat$ or pre operat$).mp	(preoperat$ or pre operat$).mp	(preoperat$ or pre operat$).mp	
chang$.tw	chang$.tw	chang$.tw	chang$.tw
evaluat$.tw	evaluat$.tw	evaluat$.tw	evaluat$.tw
reviewed.tw	reviewed.tw	reviewed.tw	reviewed.tw
prospective$.tw	prospective$.tw		
retrospective$.tw	retrospective$.tw		
baseline.tw	baseline.tw		baseline.tw
cohort.tw	cohort.tw		
consecutive$.tw			
(compare$ or compara$).tw		(compare$ or compara$).tw	(compare$ or compara$).tw
	case series.tw		

The performances of the filters are detailed in Table 6. While all the EMBASE and combined MEDLINE/EMBASE filters achieved 100% sensitivity, the two MEDLINE filters achieved a sensitivity of 99.5%, with both failing to retrieve the same article. A detailed examination of this MEDLINE record failed to find any words in the title or abstract, or any index term, that gave an indication of methodology [12].

**Table 6:** Performance of MEDLINE and EMBASE Filters

Filter	Retrieved			Sensitivity % (95% CI)	Precision % (95%CI)	Specificity % (95% CI)
				
	Reference Standard	Total	% Reduction			
**MEDLINE**						
No filter	206	1564	-	100.0	13.2 (11.5–14.9)	
Precision	205	979	37.4	99.5 (98.5–100.)	20.9 (18.4–23.4)	42.9 (40.3–45.5)
Specificity	205	972	37.9	99.5 (98.5–100.)	21.1 (18.5–23.7)	43.5 (40.4–46.6)
**EMBASE**						
No filter	191	1521	-	100.0	12.6 (10.9–14.3)	
Precision	191	1065	30.0	100.0	17.9 (15.6–20.2)	35.3 (32.7–37.9)
Specificity	191	1016	33.2	100.0	18.8 (16.4–21.2)	38.0 (35.1–39.8)
**MEDLINE/EMBASE**						
No filter	217	1959	-	100.0	11.1 (9.7–12.5)	
Precision	217	1298	33.8	100.0	16.7 (14.7–18.7)	37.9 (35.3–40.5)
Specificity	217	1266	35.4	100.0	17.1 (15.0–19.2)	39.8 (37.1–42.5)

The inclusion of each of the filters to the original search strategies improved precision. For MEDLINE, precision increased from 13.2% to 20.9% or 21.1% depending on the filter used, and was highest for the *Specificity Terms Filter*. This pattern was also evident for the EMBASE searches (from 12.6%, increasing to 18.8% for the *Specificity Terms Filter*); and for the combined MEDLINE/EMBASE search (from 11.1% increasing to 17.1% for the *Specificity Terms Filter)*. In terms of specificity achieved, the *Specificity Terms Filter *again performed marginally better. This was a consistent finding for both databases separately as well as in combination. The filters substantially reduced the number of articles retrieved varying from 30.0% to 37.9%. Once again the *Specificity Terms Filter *showed marginally superior performance, reducing the number retrieved from 1564 to 972 for Medline and from 1521 to 1016 for Embase.

Given the consistent finding that the *Specificity Terms Filter *performed marginally better than the *Precision Terms Filter*, the former was chosen as the preferred filter and was tested against the validation standards.

### Validation of filters

The MEDLINE and EMBASE *Specificity Terms Filters *were run against the two validation standards and the results are presented in Table 7. For the PRK set, there were 39 studies in total: all were indexed in MEDLINE with 33 in EMBASE. The MEDLINE filter retrieved 100% while the EMBASE filter achieved 97.0%, failing to retrieve one record. The combined filter, however, retained 100% of the validation standard. The tonsillectomy set included 30 studies in total, 27 of which were indexed in each database. While the combined filter retrieved all 30 studies, the separate filters missed four records from MEDLINE and one from EMBASE.

**Table 7:** Performance of Specificity Filters against Validation Sets.

	MEDLINE		EMBASE		MEDLINE/EMBASE Deduplicated	
	No filter	Filter	No filter	Filter	No filter	Filter

**PRK**						
Reference Standard	39	39	33	32	39	39
Total retrieved	682	452	503	393	792	567
Reference standard retrieved (%)		100.0		97.0		100.0
Reduction in retrievals (%)		33.7		21.9		28.4
**Tonsillectomy**						
Reference Standard	27	23	27	26	30	30
Total retrieved	1466	886	1213	852	1712	1196
Reference standard retrieved (%)		85.2		96.3		100.0
Reduction in retrievals (%)		39.6		29.8		30.1

The six excluded records were examined [13-18]. One of the MEDLINE records had no abstract and another had no appropriate indexed term but included the phrase *case control *in the abstract. The other MEDLINE records included the text word *post-tonsillectomy *and were indexed with the term *Postoperative complications*. For the two EMBASE records, the excluded PRK record contained the textword *consecutive *while the excluded tonsillectomy record contained the text word *retrospective *and was inaccurately indexed with the term *Retrospective study *– since all the tonsillectomy studies had been assessed as being prospective in design.

Both MEDLINE and EMBASE filters resulted in a substantial reduction in the number of retrievals but was greatest for the MEDLINE filter (PRK: 33.7% vs 21.9% and tonsillectomy: 39.6% vs 29.8%). Using the filters in combination in a multifile search resulted in a reduction of 28.4% (from 792 to 567) in retrievals for the PRK search and 30.1% (from1712 to 1196) for the tonsillectomy search.

## Discussion

In a review of methodological search filters, Jenkins describes the identification of a gold or quasi-gold standard as the set of relevant records against which filters are assessed [19]. Typically this is achieved by hand searching a set of journals. Our study, however, was carried out in conjunction with a health technology assessment, where we used the results of screening titles and abstracts from a subject-only search. We therefore recognise that this may have failed to pick up some relevant records because either the subject search failed to retrieve them; because they were missed during the screening process due to error; or because the title and abstracts failed to provide sufficient information or gave misleading information. For these reasons we called our set a reference standard, rather than the gold standard.

Ideally the gold or reference standard should be representative of all indexed records to ensure that the resulting filter has generalizability. This can be a limitation of using a gold standard based on hand searching a particular set of journals [19]. While our reference standard was not limited in this way, having been derived from an electronic search of all MEDLINE and EMBASE for particular years, there may still be bias in that our set was confined to those journals that publish papers on refractive surgery. Furthermore our reference set was limited to the publication years 2000–2004 and may have benefited from better indexing in these years [19]. Some support for these limitations is evident from the performance of the filters on the validation sets. The MEDLINE and EMBASE filters, run separately, performed better on the PRK (refractive surgery and same time frame) rather than the tonsillectomy set (otolaryngology and includes earlier time period).

Reporting of studies in the titles and abstracts infrequently used explicit terms that describe study design. Terms such as *case series, cohort, observational, non-random *and *non comparative *(including variations of these terms) appeared in only a small proportion of records and hence had low sensitivity. The exception was the term *compare$ or compara$*. Terminology that was used in the abstracts was often non-explicit, giving an indication of general systematic assessment. Those retained in the filters were *evaluat$, reviewed, chang$, consecutive$ *and *preoperat$*. The use of structured abstracts, to improve explicit reporting of methodology in the abstract, would facilitate text word searching and could assist in more effective indexing.

The search filters were developed using an objective approach. The criterion for inclusion of terms was based on each term's ability to exclude irrelevant articles rather than ability to retrieve relevant ones. This approach aimed to include those terms with highest specificity or precision that in combination produced maximum sensitivity irrespective of the sensitivity of the individual terms. The performances of the *Specificity *and *Precision Terms Filters *were similar with the former being consistently marginally better in terms of overall precision and specificity for both databases separately and in combination, while sensitivity was the same. The similar performance is not unexpected given that the majority of the search terms were common to both filters. For MEDLINE 10 out of 12 terms were included in both filters while for EMBASE, 7 were the same out of 8 in the *Precision Terms Filter *and out of 9 in the *Specificity Terms Filter*.

The validation standards were pragmatically derived, comprising the included studies in two other health technology assessments. Although both sets contained a small amount of records, they were independent of the reference standard. The MEDLINE and EMBASE filters retrieved between 85.2% and 100% of the validation standards. These would have been improved with the addition of the terms *post operative *and *case control *for MEDLINE and *consecutive *and *retrospective *for EMBASE (although only prospective studies had been included) but would have reduced the precision of the searches. The one MEDLINE record that did not have an abstract or any methodological related terms would only have been retrieved if no filter had been used.

In combination however, when both MEDLINE and EMBASE were searched, 100% retrieval was achieved, for both validation standards, while reducing the number of retrievals by the same order as found for the reference standard. When comprehensive searching is required, as is usually the case for health technology assessments or systematic reviews, searching both MEDLINE and EMBASE is generally undertaken. The results of the validation would therefore suggest that the deficiencies in sensitivity of the individual filters would be considerably reduced when used in the context of multi-database searching.

The resulting filters were developed and tested on sets of studies relating to surgical interventions. With the exception of the terms *preoperat$ or pre operat$.mp*, the terms used in the filters would be applicable to other interventions and further development could widen its applicability.

## Conclusion

The preferred MEDLINE and EMBASE filters, in combination with a subject search, maintained the sensitivity of the original subject search while at the same time reducing the number of irrelevant records retrieved by 33.2–37.9%. This performance was maintained when assessed against the validations sets. This was an initial attempt using a set of 217 records and small validation sets to develop a suitable filter to identify non-randomised studies for use in research where comprehensive searches are required. Further exploration is desirable to further test this filter using a larger dataset and to adapt it for use to a non-surgical context.

## Competing interests

The author(s) declare that they have no competing interests.

## Authors' contributions

CF developed and validated the filters and drafted the manuscript; AM screened the records for the original review, assessed study design and contributed to the manuscript; JB contributed to the manuscript. All authors read and approved the final manuscript.

## Pre-publication history

The pre-publication history for this paper can be accessed here:


